# Supplementary material for: Akt and STAT5 mediate naïve human CD4+ T-cell early metabolic response to TCR stimulation
Source: Nat Commun. 2019 May 3;10:2042. doi: 10.1038/s41467-019-10023-4 (PMC6499791; doi:10.1038/s41467-019-10023-4)
Supplement: Supplementary file 1 — Supplementary Information [file 41467_2019_10023_MOESM1_ESM.pdf]

SUPPLEMENTARY INFORMATION

Immediate TCR-induced metabolic response in naïve CD4+ T-cells is mediated by Akt and STAT5

Jones et al.,

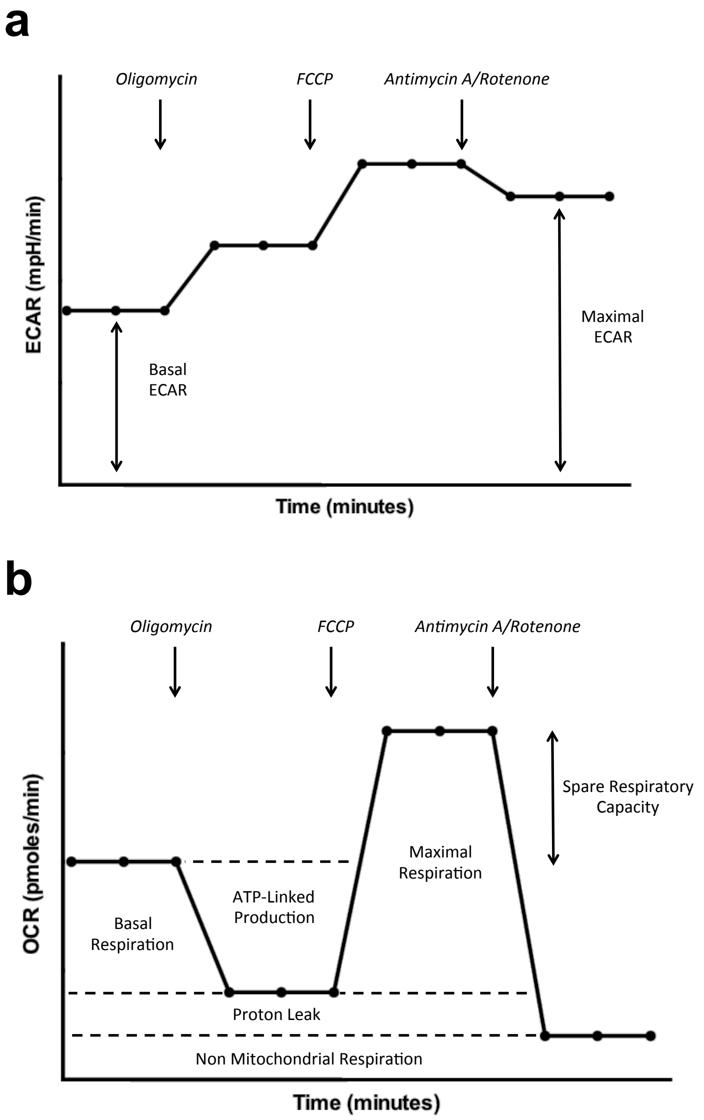

Supplementary Figure 1. Metabolic parameters measured using oxygen consumption rate (OCR) or extracellular acidification rate (ECAR) using the Seahorse XF^{e} analyser (**a**) Oxidative parameters of the mitochondria measured using OCR (pmoles/min), specifically basal, ATP-linked, maximal respiration, spare respiratory capacity, proton leak and non-mitochondrial respiration. Calculated with use of injections, oligomycin, FCCP and antimycin A/rotenone. (**b**) Glycolytic parameters measured using ECAR (mpH/min), basal and maximal ECAR levels with use of injections above.

a

**Parameter (OCR)**	**Calculation**
Proton leak	Difference between last three and oligomycin injection measurements
Non-mitochondrial respiration	Measurement after antimycin A/rotenone
Basal respiration	Initial three measurements – non-mitochondrial respiration
ATP-linked production	Basal respiration – proton leak
Maximal respiration	Difference between FCCP and antimycin A/rotenone injections
Spare respiratory capacity	Basal respiration – maximal respiration

b

**Parameter (ECAR)**	**Calculation**
Basal ECAR	Average initial three measurements
Maximal ECAR	Average last three measurements

Supplementary Table 1. Various calculations used to measure metabolic parameters. (**a**) Oxidative phosphorylation parameter calculations and (**b**) glycolytic parameter calculations. Both measured using averaged triplicate measurements.

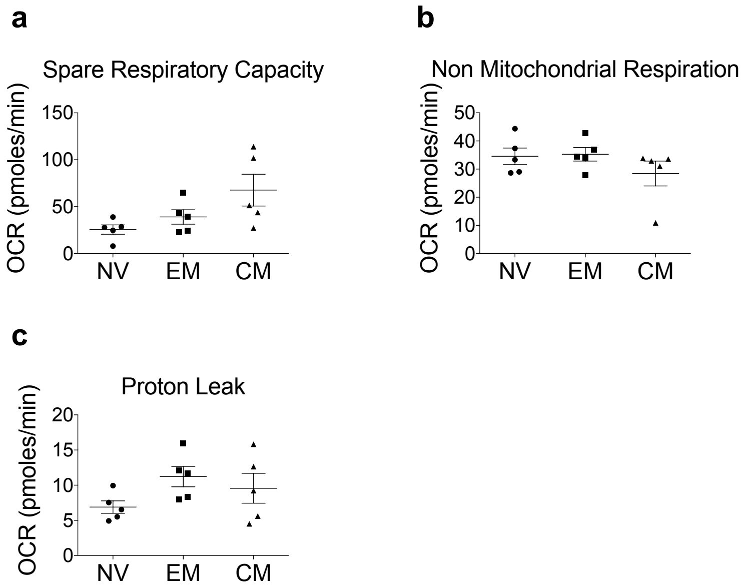

Supplementary Figure 2. Additional oxidative parameters between NV, EM and CM T cells (**a**) Spare respiratory capacity, (**b**) Non-mitochondrial respiration and (**c**) proton leak of NV, EM and CM subset populations. Data are from five independent experiments and are expressed as mean ± SEM.

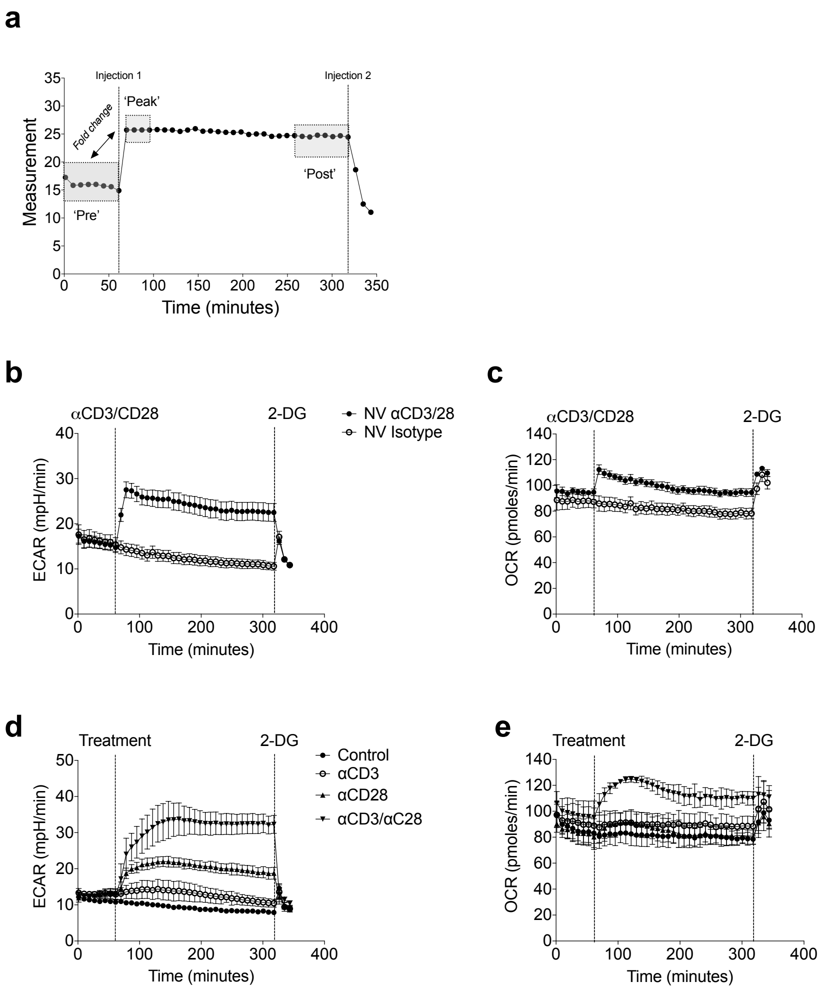

Supplementary Figure 3. NV T cells engage glycolysis and oxygen consumption upon stimulation. (**a**) Method of calculating fold change and 'pre', 'peak' and 'post' measurements. (**b**) Extracellular acidification rate (ECAR) and (**c**) oxygen consumption rate of NV T cells upon stimulation with antibodies, anti-CD3 (0.2 µg/mL) and anti-CD28 (20 µg/mL). NV T cells were subjected to anti-CD3 and anti-CD28 injection with 2-deoxy-D-glucose (2-DG; 100 mM) added at the end of the experiment to immediately arrest glycolysis. Simultaneous measurements of ECAR and OCR changes that occurred during the assay. Matched isotype controls for the activating antibodies were used and did not induce any metabolic changes. (**d**) ECAR and (**e**) OCR of NV T cells upon stimulation with antibodies, anti-CD3 (0.2 µg/mL), anti-CD28 (20 µg/mL) or a combination of anti-CD3 and anti-CD28 (0.2 and 20 µg/mL respectively). Data are from four (**b,c**) or three (**d,e**) independent experiments and are expressed as mean ± SEM.

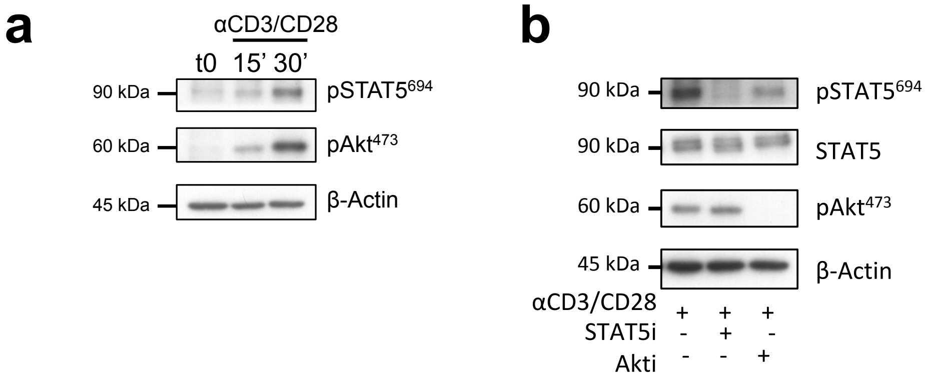

Supplementary Figure 4. STAT5 and Akt are immediately phosphorylated upon TCR ligation in NV T cells. (a) NV T cells were activated for 15 or 30 minutes with anti-CD3 (2 μg/mL) and anti-CD28 (20 μg/mL). Protein immunoblot for pSTAT5^{694}, pAkt^{473} and housekeeping β-actin. (**b**) NV T cells were activated for 30 minutes in the presence of either the STAT5 inhibitor (N′-((4-Oxo-4H-chromen-3-yl)methylene)nicotinohydrazide; 100 μM) or Akt1/2 inhibitor (10 μM). Protein immunoblot for pSTAT5^{694}, STAT5, pAkt^{473} and housekeeping β-actin. Data from one representative sample of four (**a**) or two (**b**) experiments are shown.

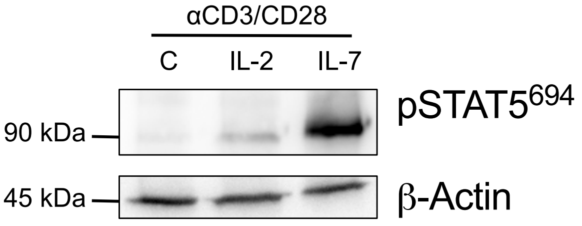

Supplementary Figure 5. The effect of gamma chain cytokines IL-2 and IL-7 on STAT5 phosphorylation. NV T cells were activated for 30 minutes with anti-CD3 (2 μg/mL) and anti-CD28 (20 μg/mL) with or without IL-2 or IL-7 (both 10 ng/mL; Miltenyi Biotech). Protein immunoblot for pSTAT5^{694} and housekeeping β-actin; data from one representative sample of two experiments are shown.

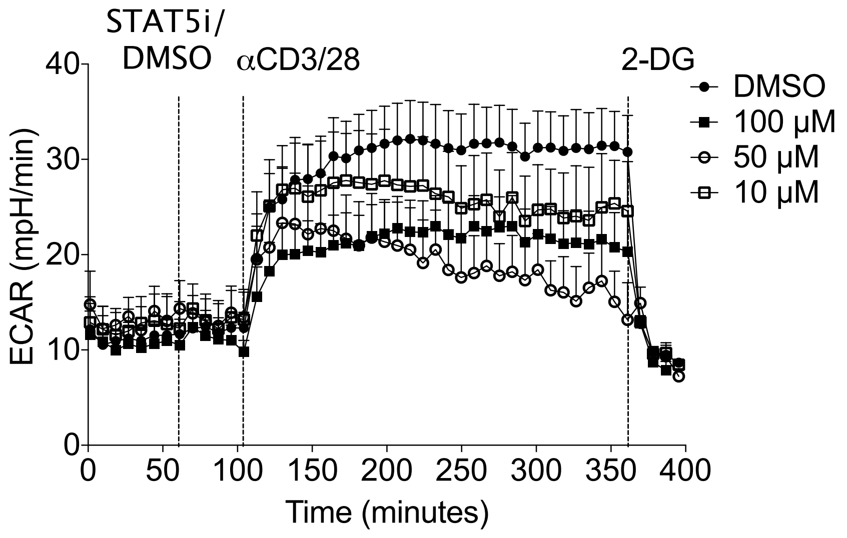

Supplementary Figure 6. Glycolytic response to dose course of STAT5 inhibitor. NV T cells were exposed to STAT5 inhibitor, N′-((4-Oxo-4H-chromen-3-yl)methylene)nicotinohydrazide at varying concentrations (10, 50 and 100 μM) before activation with anti-CD3 (2 μg/mL) and anti-CD28 (20 μg/mL) and the glycolytic response measured via ECAR. A final injection of 2-DG was used to arrest glycolysis. This experiment was repeated three to five times and data are expressed as mean + SEM.

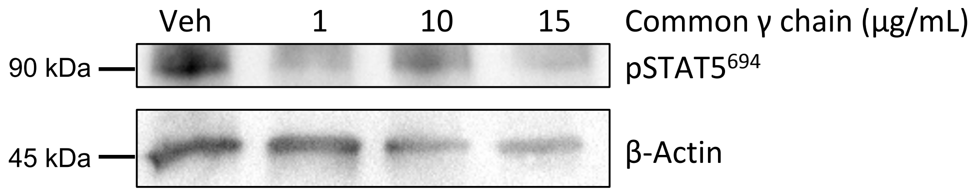

Supplementary Figure 7. The common γ chain antibody reduces IL-2 mediated STAT5 phosphorylation in NV T cells. Immunoblot of NV T cells stimulated with IL-2 (10 ng/mL; Miltenyi Biotec) and a common gamma chain antibody (1, 10 and 15 μg/mL) for 3 hours. Protein immunoblot for $pSTAT5^{694}$ and housekeeping β-actin; data from one representative sample of two experiments are shown.

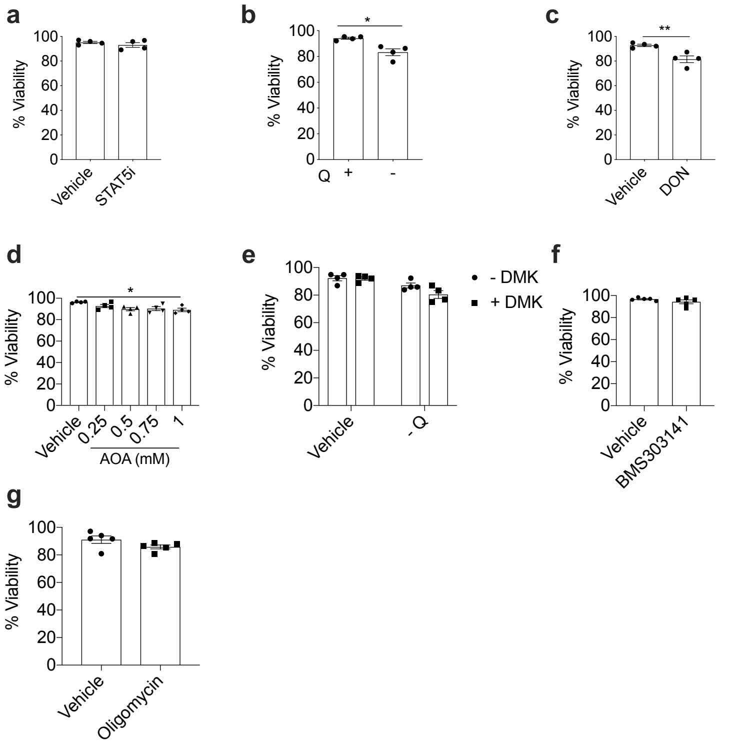

Supplementary Figure 8. The effect of DMK supplementation, oligomycin, AOA and BMS303141 inhibition on NV T cell viability. (**a**) Viability was determined by DRAQ7 (1 µM) flow cytometry of CD4+ NV T cells. Stimulated NV T cells with anti-CD3 (2 µg/mL) and anti-CD28 (20 µg/mL) were cultured for 24 h with or without (**a**) STAT5i (100 µM), (**b**) glutamine withdrawal, (**c**) DON treatment, (**d**) AOA (0.25-1 mM), (**e**) ± glutamine withdrawal ± DMK (0.3 mM), (**f**) BMS303141 (0.01-1 µM) or (**g**) oligomycin (100 nM). Data are from four independent experiments (**a-f**) or five experiments (**g**) and are expressed as mean + SEM; * $p \leq 0.05$, ** $p \leq 0.01$.

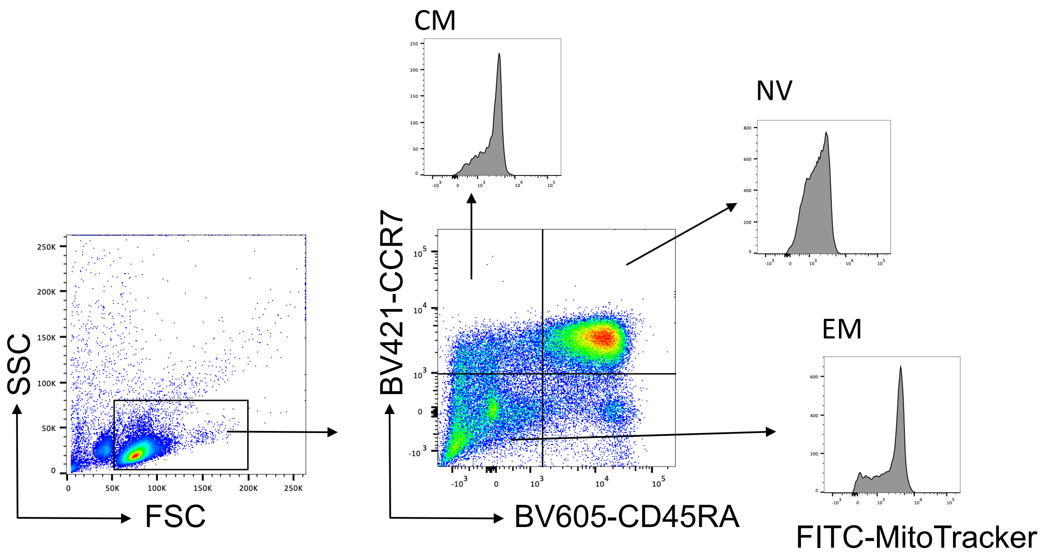

Supplementary Figure 9. Representative gating strategy. Gating strategy used to analyse MitoTracker Green content of CD4+ NV, EM and CM T cell subsets (Figure 2f).

Figure 1d.

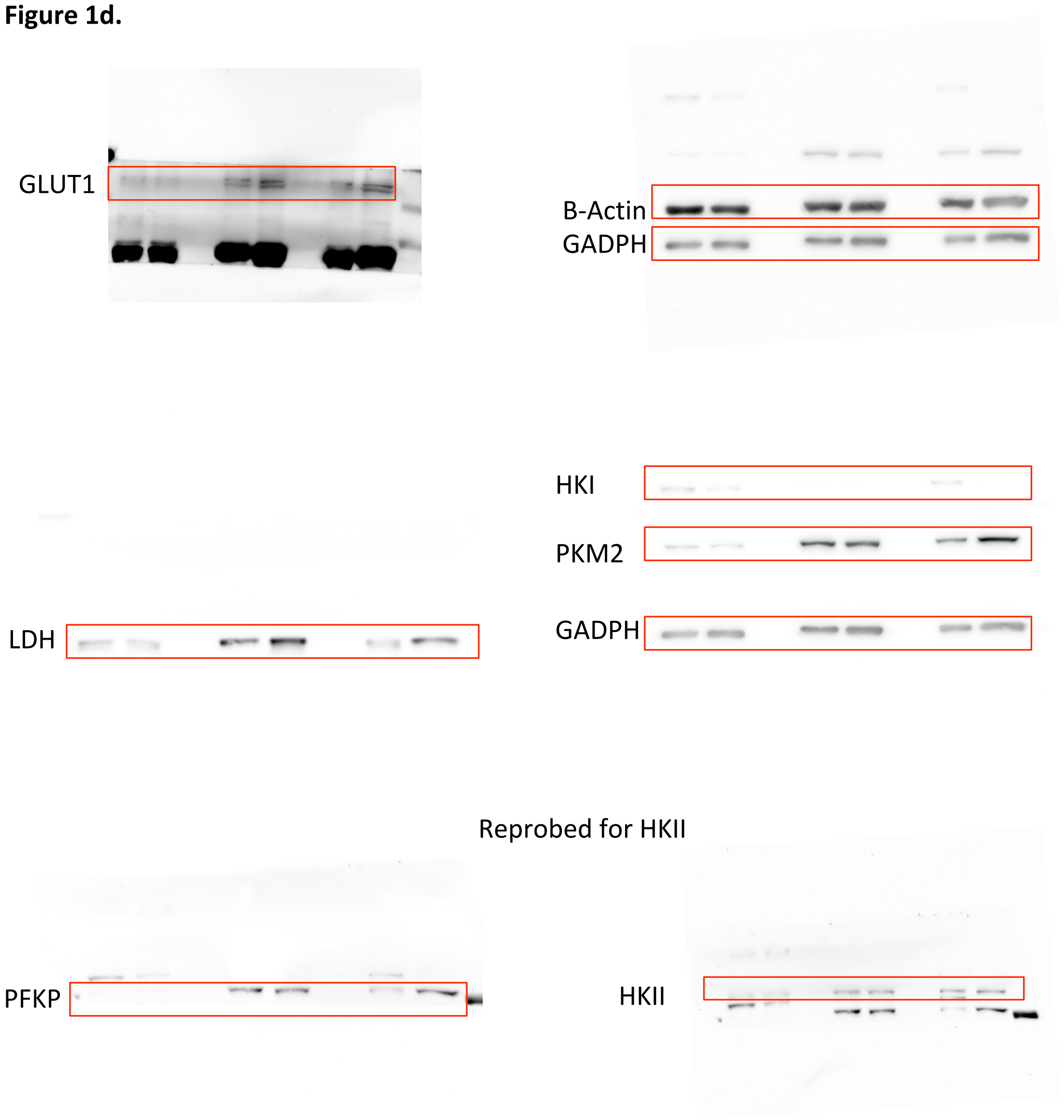
GLUT1
B-Actin
GADPH
HKI
PKM2
LDH
GADPH
Reprobed for HKII
PFKP
HKII

Figure 4a/5a

NV T cell

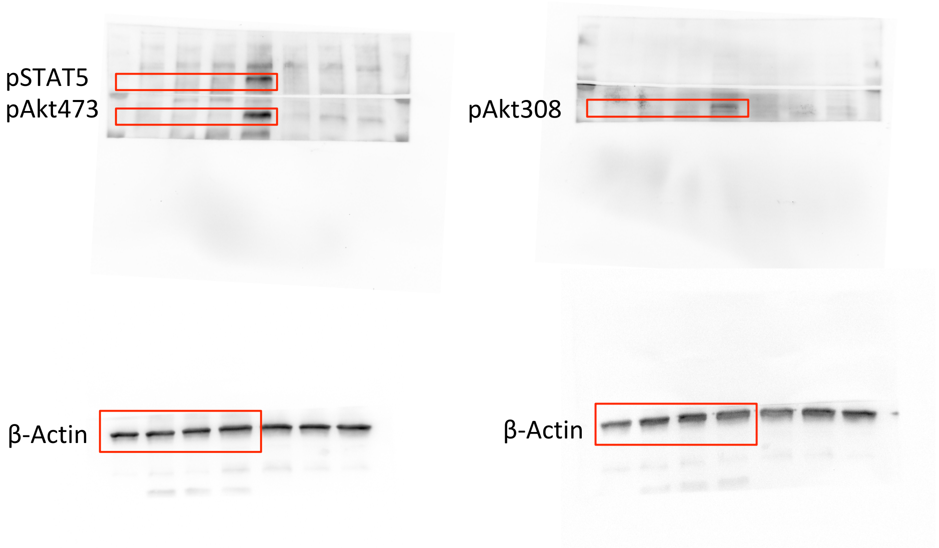

Figure 4a/5a

EM T cell

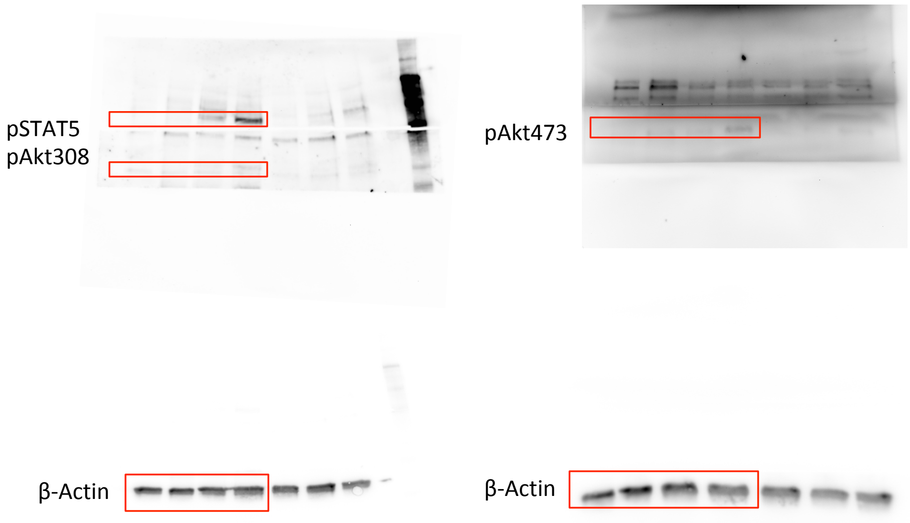

Figure 4a/5a

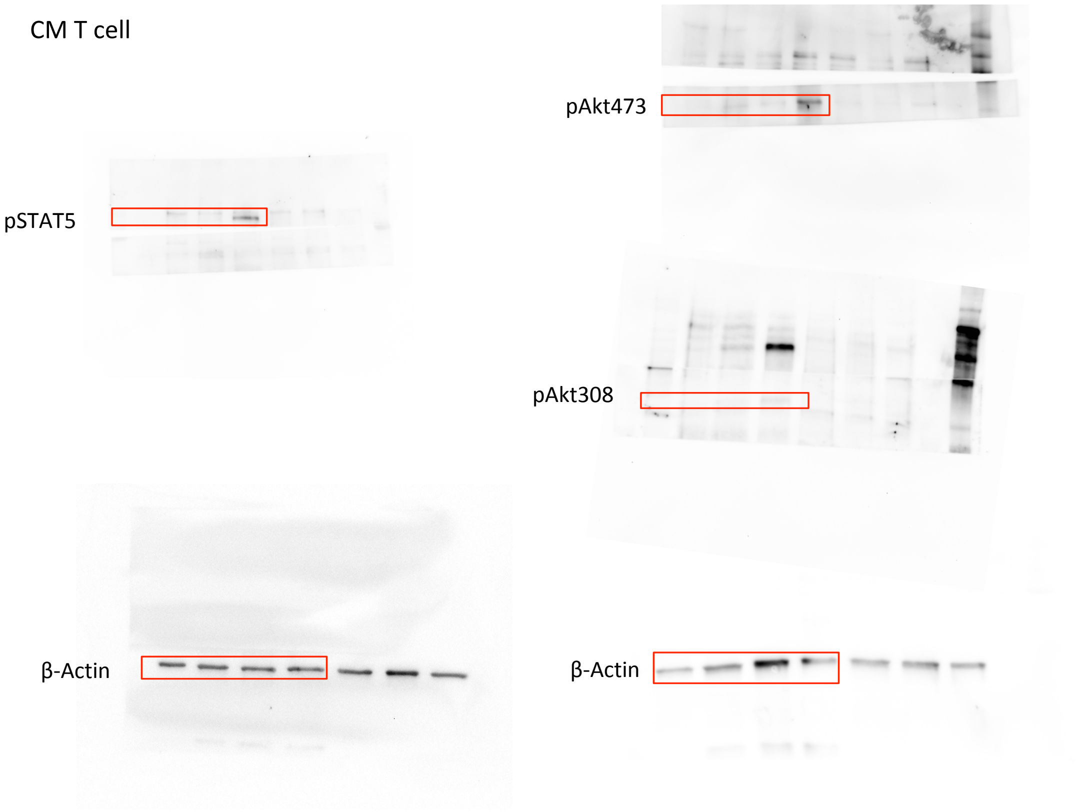

Figure 5b

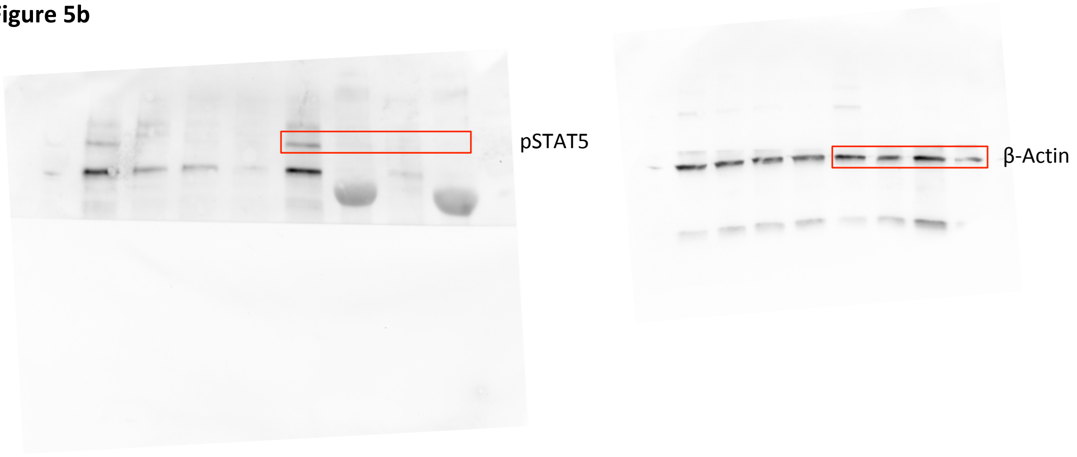

Figure 5m

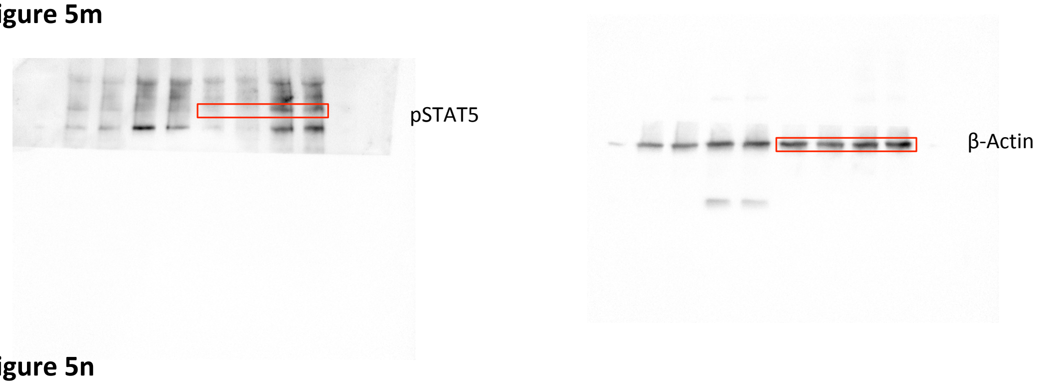

Figure 5n

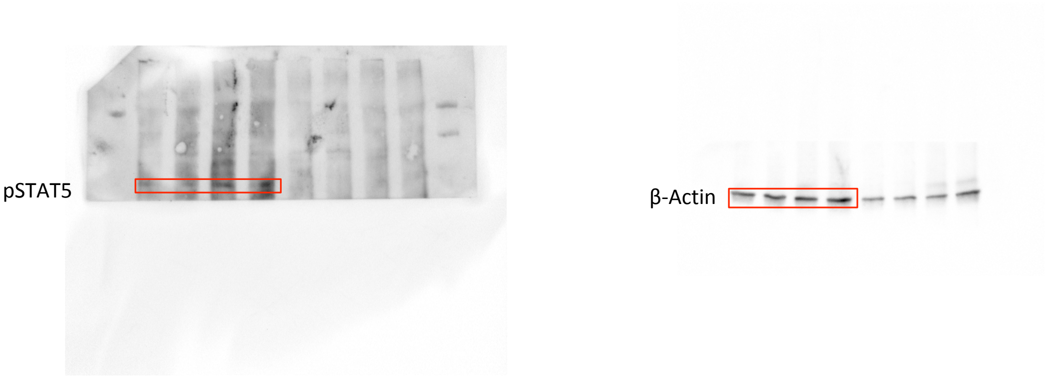

Figure 6f

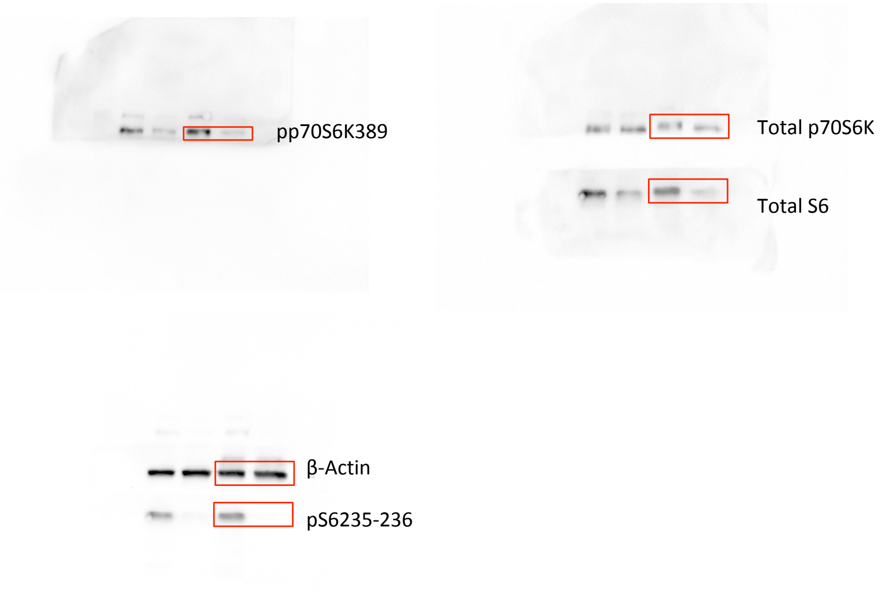

Supplemental Figure 4a

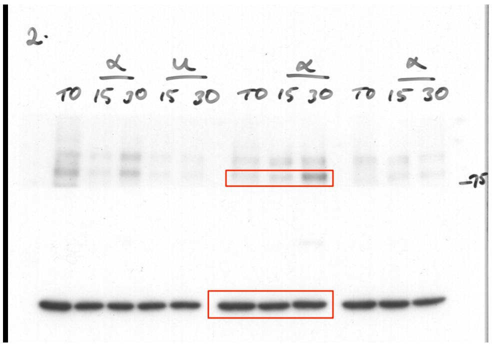

pSTAT5

β-Actin

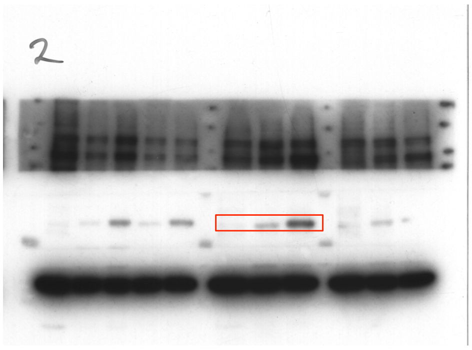

pAkt473

Supplemental Figure 4b

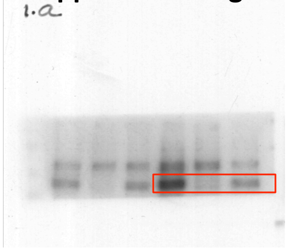

pSTAT5

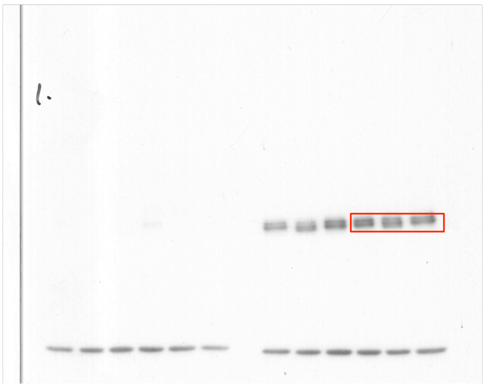

Total STAT5

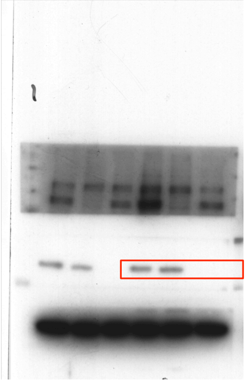

pAkt473

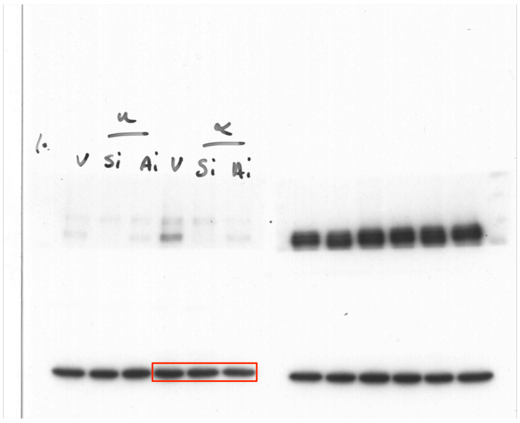

β-Actin

Supplemental Figure 5

pSTAT5

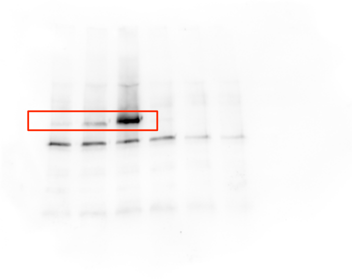

β-Actin

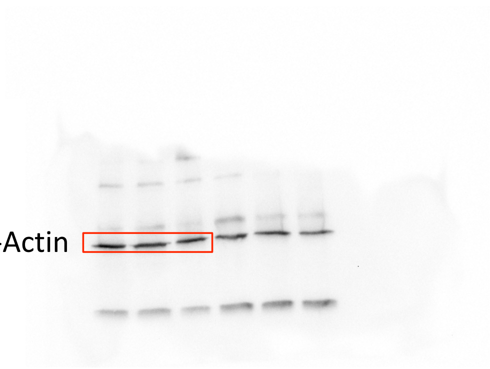

Supplemental Figure 7

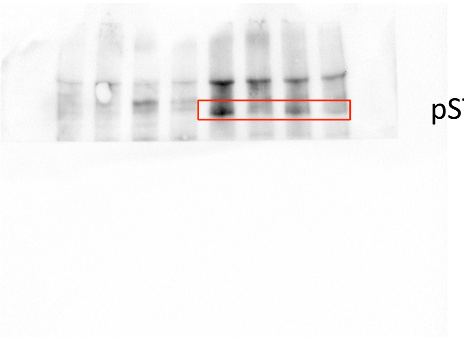

pSTAT5

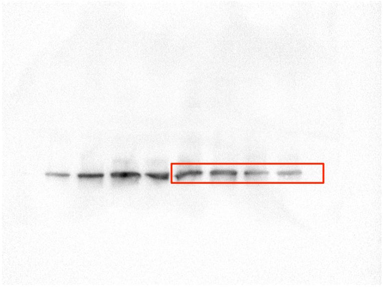

β-Actin

Supplementary Figure 10. Original uncropped immunoblots for main figures and supplementary figures.
