# Supplementary material for: Akt and STAT5 mediate naïve human CD4+ T-cell early metabolic response to TCR stimulation
Source: Nat Commun. 2019 May 3;10:2042. doi: 10.1038/s41467-019-10023-4 (PMC6499791; doi:10.1038/s41467-019-10023-4)
Supplement: Supplementary file 3 — Reporting Summary [file 41467_2019_10023_MOESM3_ESM.pdf]

nature research

Corresponding author(s): C A Thornton

Last updated by author(s): Feb 28, 2019

Reporting Summary

Nature Research wishes to improve the reproducibility of the work that we publish. This form provides structure for consistency and transparency in reporting. For further information on Nature Research policies, see Authors & Referees and the Editorial Policy Checklist.

Statistics

For all statistical analyses, confirm that the following items are present in the figure legend, table legend, main text, or Methods section.

n/a	Confirmed	
☐	☒	The exact sample size (n) for each experimental group/condition, given as a discrete number and unit of measurement
☐	☒	A statement on whether measurements were taken from distinct samples or whether the same sample was measured repeatedly
☐	☒	The statistical test(s) used AND whether they are one- or two-sided<br>*Only common tests should be described solely by name; describe more complex techniques in the Methods section.*
☒	☐	A description of all covariates tested
☐	☒	A description of any assumptions or corrections, such as tests of normality and adjustment for multiple comparisons
☐	☒	A full description of the statistical parameters including central tendency (e.g. means) or other basic estimates (e.g. regression coefficient) AND variation (e.g. standard deviation) or associated estimates of uncertainty (e.g. confidence intervals)
☐	☒	For null hypothesis testing, the test statistic (e.g. F, t, r) with confidence intervals, effect sizes, degrees of freedom and P value noted<br>*Give P values as exact values whenever suitable.*
☒	☐	For Bayesian analysis, information on the choice of priors and Markov chain Monte Carlo settings
☒	☐	For hierarchical and complex designs, identification of the appropriate level for tests and full reporting of outcomes
☒	☐	Estimates of effect sizes (e.g. Cohen's d, Pearson's r), indicating how they were calculated

Our web collection on statistics for biologists contains articles on many of the points above.

Software and code

Policy information about availability of computer code

Data collection	ImageLab (BioRad) V 5.2.1, FACS Diva Software, Seahorse Wave Software
Data analysis	GraphPad (Prism) V8, Mass spectrometry data: ChemStation, Immunoblot and confocal data: Fiji ImageJ, Flow cytometry data: FlowJo V10

For manuscripts utilizing custom algorithms or software that are central to the research but not yet described in published literature, software must be made available to editors/reviewers. We strongly encourage code deposition in a community repository (e.g. GitHub). See the Nature Research guidelines for submitting code & software for further information.

Data

Policy information about availability of data

All manuscripts must include a data availability statement. This statement should provide the following information, where applicable:

- Accession codes, unique identifiers, or web links for publicly available datasets
- A list of figures that have associated raw data
- A description of any restrictions on data availability

All data are available upon request and can be found within the manuscript and supplementary information

Field-specific reporting

Please select the one below that is the best fit for your research. If you are not sure, read the appropriate sections before making your selection.

☒ Life sciences ☐ Behavioural & social sciences ☐ Ecological, evolutionary & environmental sciences

Life sciences study design

All studies must disclose on these points even when the disclosure is negative.

Sample size	Sample size did not need to be determined for this study.
Data exclusions	No data was excluded.
Replication	All experiments were replicated as described in the figure legend
Randomization	This was not needed for this study.
Blinding	N/A

Reporting for specific materials, systems and methods

We require information from authors about some types of materials, experimental systems and methods used in many studies. Here, indicate whether each material, system or method listed is relevant to your study. If you are not sure if a list item applies to your research, read the appropriate section before selecting a response.

Materials & experimental systems

n/a	Involved in the study	
☐	☒	Antibodies
☒	☐	Eukaryotic cell lines
☒	☐	Palaeontology
☒	☐	Animals and other organisms
☐	☒	Human research participants
☒	☐	Clinical data

Methods

n/a	Involved in the study	
☒	☐	ChIP-seq
☐	☒	Flow cytometry
☒	☐	MRI-based neuroimaging

Antibodies

Antibodies used

Cell Signalling
Glucose transporter 1 (GLUT1; 12939)
hexokinase I (HKI; 2024)
hexokinase II (HKII; 2867)
glyceraldehyde-3-phosphate dehydrogenase (GAPDH; 5174)
phosphofructokinase (PFK; 8164)
pyruvate kinase (PKM2; 4053)
lactate dehydrogenase (LDHA; 3582)
phospho-STAT5 Tyr694 (9351)
total STAT5 (9363)
phospho-Akt Thr308 (9275) and Ser473 (9271)
phospho-S6 ribosomal protein (Ser235-236; 4858)
total S6 ribosomal protein (2217)
phospho-p70 S6 kinase (Thr389; 9234)
total p70 S6 kinase (2708)
Abcam
β-actin (8226)

anti-CD4 AlexaFluor®647 (mIgG2b, clone OKT4, BioLegend 317422)
anti-CD45RA Brilliant Violet 605 (mIgG2b, clone HI100, BioLegend 304134)
anti-CD45RO FITC (mIgG2a, clone UCHL1, BioLegend 304242)
anti-CD197 Brilliant Violet 421 (mIgG2a, clone G043H7, BioLegend 353208)
PE-labelled CD69 (mIgG1, FN50, BioLegend 310906)
anti-CD3 (HIT3a, BioLegend 300314)
anti-CD28 (CD28.2, BioLegend 302923)

common γ chain antibody (R&D Systems)

Validation

All antibodies are commercially available

Human research participants

Policy information about studies involving human research participants

Population characteristics	The study was performed on a population of healthy adults aged 18 - 70 years old and included men and women. Participants were excluded if they had an immune-mediated disease, cancer in the past 5 years or had current/recent symptoms of viral or other infection. Participants using medication, such as statins, with immune response modifying effects, were also excluded. All samples were collected between 0800 and 1200.
Recruitment	Participants were recruited from the staff and student populations at Swansea University, Wales UK. Potential participants responded to ethics committee approved advertising by contacting the local clinical research facility. The clinical research facility oversaw recruitment through informed written consent in response to an ethically approved participant information sheet that explained the study.
Ethics oversight	This project was approved by Wales Research Ethics Committee 6 (approval 13/WA/0190) which is a committee within the Health Research Authority structure within the UK and equivalent to Institutional Review Board in USA.

Note that full information on the approval of the study protocol must also be provided in the manuscript.

Flow Cytometry

Plots

Confirm that:

☒ The axis labels state the marker and fluorochrome used (e.g. CD4-FITC).

☒ The axis scales are clearly visible. Include numbers along axes only for bottom left plot of group (a 'group' is an analysis of identical markers).

☒ All plots are contour plots with outliers or pseudocolor plots.

☒ A numerical value for number of cells or percentage (with statistics) is provided.

Methodology

Sample preparation	Methods: Flow cytometry
Instrument	FACS Aria I
Software	FACS Diva software acquisition, FlowJo V 10 analysis
Cell population abundance	Purity was in excess of 90% for each CD4+ T cell subset analysed post autoMACS separation
Gating strategy	Gating strategies are present in the supplementary information.

☒ Tick this box to confirm that a figure exemplifying the gating strategy is provided in the Supplementary Information.
